# Cultural Diversity and Mental Health: Considerations for Policy and Practice

**DOI:** 10.3389/fpubh.2018.00179

**Published:** 2018-06-19

**Authors:** Narayan Gopalkrishnan

**Affiliations:** Social Work, James Cook University, Cairns, QLD, Australia

**Keywords:** culture, cultural diversity, mental health, mental illness, cultural partnerships

## Abstract

The purpose of this paper is to explore some of the key considerations that lie at the intersection of cultural diversity and mental health. Mental health providers and professionals across the world have to work with clients that are often from cultures other than their own. The differences in cultures have a range of implications for mental health practice, ranging from the ways that people view health and illness, to treatment seeking patterns, the nature of the therapeutic relationship and issues of racism and discrimination. This paper will excavate some of these considerations with a view to raising possible ways in which mental health systems and professionals can engage across cultures more equitably and sustainably.

## Introduction

Culture is a broad and vexed term that can be defined in a range of ways, depending on the field of study and the perspective of the person using the term. As Tribe (1) argues, it is a multi-layered concept influenced by a range of issues such as gender, class, religion, language, and nationality, just to name a few. Giddens (2), from a sociological perspective, presents culture as the set of values that the members of a given group hold and includes the norms they follow and the material goods that they create. For the purposes of this paper we are using the term in the context of ethnic identity, or the multidimensional set of ascriptive group identities to which religion, language, and race (as a social construct) belong and all of which contribute to a person's view of themselves (3–5).

Culture, in itself, is impacted on by the broader context of social norms and social issues. For example, many refugees, and migrants in Western countries work at the lower ends of the labor market, with lower pay and higher levels of casualization than the general population (6). As such they experience much higher levels of income inequality within these countries, inequalities that in turn impact adversely on their health and wellbeing (7). While acknowledging the key impacts of these larger social issues, and recognizing the need for broad and collective responses to their impacts on cultural groups, this paper will focus on the impacts that culture has on mental health so as to excavate the key considerations for policy and practice within this context.

Much of the theory and practice of mental health, including psychiatry and mainstream psychology, have emerged from Western cultural traditions and Western understandings of the human condition. Notions of Cartesian dualism of body and mind, positivism, and reductionism have been central to the development of mainstream mental health systems as they are widely implemented today (8, 9). While these relatively monocultural understandings of mental health have provided powerful conceptual tools and frameworks for the alleviation of mental distress in many settings, they have also been very problematic when applied to the context of non-Western cultures without consideration of the complexity that working across cultures brings with it (10, 11). Tribe [(1), p. 8] suggests that Western cultural approaches to health tend to be “predicated on a model that focuses on individual intrapsychic experience or individual pathology, while other traditions may be based more on community or familial processes.” From issues of over-representation of particular cultural groups in mental health facilities to research that excludes cultural groups and includes others, there are a number of areas at the intersection of mental health and culture that need to be considered by the mental health professional if they wish to effectively engage with all of the people that they work with (1, 12).

Cultural diversity across the world has significant impacts on the many aspects of mental health, ranging from the ways in which health and illness are perceived, health seeking behavior, attitudes of the consumer as well as the practitioners and mental health systems. As Hernandez et al. [(13), p. 1047] suggest “culture influences what gets defined as a problem, how the problem is understood and which solutions to the problem are acceptable.” Many of these considerations in cultural diversity and mental health will be explored in the rest of the paper, an exploration of importance in that while it may point to areas that need to be strengthened, it will also point to opportunities that exist where new forms of engagement could be explored and the needs of people of diverse cultures could be met more effectively and sustainably.

## Key considerations

Hechanova and Waeldle (14) suggest that there are five key components of diverse cultures that have implications for mental health professionals. While the authors make their arguments in the specific context of disaster situations in Southeast Asia, their comments provide a framework to begin the discussion of cultural diversity and mental health. The first element that they identify is *emotional expression* where some cultures may identify that lack of balance in expression may lead to disease. Further, this may be reflected in a perception that talking about painful issues would lead to further painful feelings (p. 33). This reluctance to utilize talking therapies is evidenced in other research especially with refugees from Africa and from Southeast Asia (5). The second element is *shame* which Hechanova and Waelde (14) argue is one of the reasons why Asians are slow to access professional therapists. Shame may play a key part in this context because of the significant role that family plays in the lives of Asian individuals with mental health issues (5). The third element that they discuss is *power distance* or the large differences in power that may exist in Asian countries between therapist and which may have implications in terms of the autonomy or lack thereof in the therapeutic relationship. Fourth they discuss the nature of *collectivism* and its impact as a supportive factor to resilience and coping. And finally, they discuss *spirituality and religion* from the point of view of attribution as well as in terms of coping with disease [(14), p. 34]. These five factors are useful to further excavate some of the considerations that we explore in this section.

There is an extensive body of research literature that emphasizes the fact that health and illness are perceived differently across cultures (8, 15–18). Cultural meanings of health and illness have “real consequences in terms of whether people are motivated to seek treatment, how they cope with their symptoms, how supportive their families and communities are, where they seek help (mental health specialist, primary care provider, clergy, and/or traditional healer), the pathways they take to get services, and how well they fare in treatment” [(19), p. 26]. To begin with, the *perceptions of* etiology of disease can be very different across cultures. Helman (20) presents us with a framework of views of illness causality that may be at the individual level or situated in the natural world or in the social world, and argues that each cultural group views these differently. Some cultures may ascribe the onset of disease to possession by spirits, the “evil eye,” black magic or the breaking of taboos, which then places the rectification of the problem within the purview of traditional healers, elders, or other significant people in the community. Religion and spirituality play a key part in these perceptions by juxtaposing hardship with a higher order good and the solutions are accordingly sought within the purview of these systems (14). Examples of these would be the healing temples in India or other religious pilgrimage sites across the world that are visited every day by thousands of people experiencing mental health issues.

Diverse views in terms of etiology are also central to the large traditional health systems in countries like India and China. For example, the disease factors in Traditional Chinese Medicine are often ascribed to a lack of balance between pathogenic factors of Yin and Yang. In Ayurveda, the major traditional healing system in India, mental health may be perceived to be a product of karma or one's actions, vayu or air, and swabhave or one's nature (5, 16). An important factor in both these healing systems is that the demarcation between body and mind is not emphasized, and the patient is treated as a whole, and in the context of his/her external environment (21, 22). It is important to note that people from diverse cultures may not make the same distinction between issues of the body and the mind as in Western therapeutic systems. In their research with Afghan refugees in the Netherlands, Feldmann et al. (23) found that the participants made no distinction between mental and physical health. This is very unlike Western biomedicine, which has traditionally taken a reductionist approach that clearly separates the body and the mind. Research in more recent times clearly points to the interrelationship between body and mind, and areas of study such as psychosomatic medicine and psychoneuroimmunology have provided substantial evidence that methods working with a composite of the body and mind in the context of the environment are more likely to be effective than dualistic and reductionist approaches (17). In the context of cultures that are already familiar with these integrative approaches, we would argue that there is potential for new approaches to working with distress, whether of the body or the mind.

Cultures vary also in terms of how they *seek treatment* from mainstream Western health system. Biswas et al. (15) argue that those seeking help from mainstream health systems in India tended to present more often with somatic symptoms whereas those in the United States tended to present more with cognitive based symptoms. Further, research in High Income Countries (HICs) like Australia, Canada and the United States emphasizes that diverse cultures in these countries tend to seek help much later than those from the majority community and many of them tend to present in acute stages of mental distress (12, 17). One of the reasons for this can be the nature of shame as discussed in some of the research with migrants and refugees in HICs as well as with general populations in Low and Middle-Income Countries (LMICs) in Asia and Southeast Asia. Hampton and sharp (24) have explored the nature of shame quite comprehensively using a framework of external, internal and reflective shame to argue that mental health systems, professionals, and researchers need to recognize and mediate the effects of shame on individuals from diverse cultures if they wish to ensure effective management of mental health issues. Hechanova and Waedle (14) suggest that shame related reasons for low access to mental health systems could be due to several reasons. The first possibility is about the desire to protect the family reputation and their own dignity. The second relates to the possibility that the mental health professional would see them as “crazy,” similar to the notion of external shame, and finally that the person may be reluctant to open up to strangers, due to a number of factors such as fears of “loss of face,” lack of trust, or the fear of revisiting painful events (17, 25, 26). Research indicates that talking therapies may not be the most useful form of intervention among many cultural groups. The National Child Traumatic Stress Network in the United States argues that “talking about painful events may not be experienced as valuable or therapeutic by refugees from societies where psychological models are not hegemonic” (27). This perception of talking therapies in turn raises the possibilities of more effective utilization of movement-based therapies, expressive therapies, online therapies (28).

*Stigma* can play a key role in terms of variations in treatment-seeking. Stigma can be viewed as a “mark of shame, disgrace or disapproval which results in an individual being rejected, discriminated against, and excluded from participating in a number of different areas of society” [(29), p. 16]. Stigma around depression and other mental illness can be higher in some cultural groups and often is a major barrier to people from diverse cultures when accessing mental health services (12, 15). Stigma can cause people to feel so ashamed that they hide their symptoms and do not seek treatment until the issues becomes acute (19). Stigma can be examined from a range of related issues such as the perceptions of etiology as well as notions of shame and levels of interdependence in the community (20, 24). In the context of Low and Medium Income Countries, these issues become even more significant as the family is often the only safety net that individuals have. Where government safety nets are minimal or do not exist, lack of support from the family due to perceptions of stigma can lead to total neglect of a person with mental health issues (11).

Treatment seeking is also very closely linked to the *historical context* of cultural groups. This is of special note in in HICs with a colonial past. First Nations People in countries like the United States, Canada, Australia and New Zealand struggle with endemic mental health issues that can be closely linked to histories of dispossession, oppression and intergenerational trauma (30, 31) and as Nelson and Wilson [(32), p. 93] argue, carry a “disproportionate burden of mental and physical illness”. Gone (33) uses the term “Historical Trauma” to argue that the trauma commonly experienced among First Nations People is complex, experienced more as a collective phenomenon, is cumulative and is inter-generational in its impacts. The severity and complexity of the mental health issues experienced with First Nations Peoples can also be exacerbated by the fact that in these countries, mental health professionals may be seen as part of the problem, both from a historical perspective as well as in their roles within the State (30). These perceptions would lead to reduced utilization of mental health services as well as later and more acute-stage presentations. Similarly, research with African-American and Latino communities in the US also raises issues of mistrust of clinicians as a product of historical persecution as well as current issues of racism and discrimination (19). The challenges that this discussion on the historical perspective raises is around mental health systems that can work in more collaborative and power-sharing ways, and that work deliberately toward empowering the communities that they work with.

*Racism and discrimination* impact quite dramatically on many diverse cultural groups. While older forms of racism were ideologies that supported the notion of biological “races” and ranked them in terms of superior and inferior, these have since been superseded by newer forms of racism that are built on more complex notions of cultural superiority or inferiority (34). Besides the negative attitudes and beliefs that are implicit in all forms of racism, they also lead to discrimination and differential treatment of individuals of some cultural groups. The experience of racism can lead to social alienation of the individual, a fear of public spaces, loss of access to services, and a range of other effects that in turn impact adversely on the mental health of the affected individual (12). Williams and Mohammed [(35), p. 39], based on their systematic literature review, argue that “the consistency of an inverse association between discrimination for an increasingly broad range of health outcomes, across multiple population groups in a wide range of cultural and national contexts is impressive, and lends credibility to the plausibility of perceived discrimination as an important emerging risk factor for disease.” In the present hostile environment to Muslims in many HICs, women who dress in a way that identifies them clearly with Islam can especially bear a significant amount of individual and institutionalized racism and discrimination, with significant impacts on their mental health (36). Discrimination is also one of the major barriers to Aboriginal Peoples accessing mental health services, especially when the service is within a non-Aboriginal mental health setting (37).

As part of the discussion of racism and discrimination, notions of mainstream bias and the stereotyping of cultural groups in healthcare need consideration. The history of working with diverse cultural groups in healthcare in High Income Countries has numerous examples of stereotyping of specific cultural groups leading to interventions that are often inadequate or inappropriate (38, 39). The very concepts of normality and abnormality in Western therapeutic approaches are embedded in cultural constructions that cannot be easily generalized across cultures (27). These can lead to situations where “health practitioners overlook, misinterpret, stereotype, or otherwise mishandle their encounters with those who might be viewed as different from them in their assessment, intervention, and evaluation-planning processes” [(40), p. 7]. Of particular concern here is the overdiagnosis of particular cultural groups with particular mental disorders as in the case of the overdiagnosis of schizophrenia in African American communities (19, 39).

*Coping and resilience* are other areas of consideration in the context of cultural diversity and mental health. Coping styles refer to the ways in which people cope with both everyday as well and more extreme stressors in their lives, including mental health related stressors. The US Surgeon General [(19), p. 28] posits that a better understanding of the ways diverse cultural groups cope with adversity has “implications for the promotion of mental health, the prevention of mental illness, and the nature and severity of mental health problems.” Aldwin (41) suggests that cultural groups can show major differences in terms of the types of stressors that they experience, and how they assess these stressors. Different cultures may place stressful events differently as normative, or something that most people in that culture will experience, such as coming-of-age rituals. Further they will allocate social resources differently, leading to diverse experiences of these stressors. And finally, they may assess stressors differently, such as in terms of breaking of taboos or other cultural norms (p. 567). This diversity in terms of dealing with stressors can be both a protective factor and a risk factor. Hechanova and Waelde (14) suggest that, in collectivist cultures, healing is a product of interdependence and that the health of the group is at least as important to the individual as his or her own health.

Closely associated with coping, resilience is the ability to do well despite facing adversity, and is often discussed in the context of traits and characteristics of individuals. Kirmayer et al. (42) argue that the psychological approaches to resilience have emphasized individual traits rather than the systemic or ecological roots of resilience. They go on to suggest that, in the context of the Aboriginal Peoples of Canada, resilience is embedded in cultural values, renewed cultural identity, revitalized collective history, language, culture, spirituality, healing, and collective action. As discussed earlier, collectivist cultures can play a key role as both a protective factor and a risk factor in issues of mental health. In many cultural groups, *the family* can be very involved in all aspects of a person's life (43). Family factors such as supportive extended families and strong sibling relationships can act as protective factors in mental health, while perceptions of stigma, severe marital discord, breaking of norms and other such factors can be major risk factors (19, 44). Which would suggest that interventions that include cultural renewal and community and family support systems can be very useful in some or most cultural groups.

*Cultural impacts on the therapeutic relationship* are a significant factor to be considered in working with diverse cultures in mental health. The cultural context of the client and the practitioner are both central to the therapeutic relationship, a relationship that cannot work without careful consideration of the implications of cultural diversity. Ideally, both the therapist and the client would be from the same culture and some of the pitfalls can be avoided (45). However, even in these circumstances, the practitioner brings their own “professional” culture with them which can create a cultural gap with the client (19). In practice, there is a strong likelihood that therapists would be working with clients from cultures very different from their own and making assessments without linguistic, conceptual and normative equivalence, which could lead to many errors in service provision decision (45). Some of the issues of overdiagnosis of certain cultural groups with particular mental disorders as mentioned earlier may find its roots with this lack of equivalence in assessment (39). Still further considerations involve the concept of *culture as language* (46). Language is central to any culture and to cultural understanding, and yet in HICs such as Australia the therapist and the client may not even share the same language. While many High Income Countries have policies in place to ensure that appropriate interpreters are used in such circumstances, an endemic problem of non-utilization of interpreters continues (12).

*Society as a patient* is a term that Marsella (45) uses to point out that not all problems are located within the individual, and that the patient's well-being or lack thereof is often a product of the impacts of the external environment. This is particularly the case with migrants and refugees or Indigenous populations in HICs who may experience racism, discrimination, and attendant marginalization (30, 35). Marsella goes on to argue for mental health professionals who work across cultures to take up the roles of social activists and challenge some of the societal contexts that are impacting on their clients (2011). This societal context also involves globalization and the rapid change of systems and cultures. Globalization is not a new process but the last 100 years has seen a rapid increase in global networks, increased velocity of global flows and increased depth of global interconnectedness (47). Culture has been impacted by these global flows with the increasing domination of notions of individualism, materialism, and social fragmentation, and where “well-being may be a collateral casualty of the economic, social and cultural changes associated with globalization” [(48), p. 540]. The loss of social networks as protective factors can be very significant in terms of increasing levels of distress in culturally diverse communities such as refugees and migrants in HICs (49). Traditional healers and healing systems are being replaced by Western systems that can suffer from inadequate resourcing and may be culturally inappropriate (50). All of which points to the need for ways forward that build on these diminishing resources and strengthen the capacity of individuals and communities toward better mental health outcomes. Some possible future directions are discussed in the next section.

## Ways forward

Mainstream mental health systems are increasingly acknowledging the intersection of cultural diversity. As an example, the provision of the *cultural formulation interview* in the DSM-5 is a positive step especially as it seeks to explore cultural identity, conceptualization of illness, psychosocial stressors, vulnerability, and resilience as well as the cultural features of the relationship between the clinician and the patient (51). However, this is just one tool in the larger picture and cannot mean anything without more radical changes in systems and practices. Much of the literature in the field points to the need for holistic health services that incorporate the total context in which health and illness are experienced (12, 44, 52). Some suggestions involve the integration of mental health services with primary health care as a way of getting past some of the stigma and discrimination issues (19, 25). As Ng et al. [(53), p. 44] posit in the context of Low and Medium Income Countries “integrating mental health services into primary health care is a highly practical and viable way of closing the mental health treatment gap in settings where there are resource constraints.” Which is not to say that the same does not apply to the High-Income Countries like Australia where effective mental health responses in many Indigenous communities continue to be an unmet goal (54). More recent approaches such as the *biopsychosocial* and the *recovery* approaches in mental health or renewed calls for *medical pluralism* also offer new opportunities to work with people in a more holistic way (55, 56).

Fernando [(11), p. 555] suggests that “[m]ental health development, like development in any other field, must start by tapping into what people in any location currently want and value.” One of the ways that needs to exploration more systematically is the possibility of integrating *positive resources in the community* into the provision of mental health services. Marsella (45) argues that community-based ethno-cultural services are a positive resource in the community that can provide an essential function in working with mental health issues in diverse cultural groups. Further, he argues that the development of a strong social support and community-based network must be intrinsic to the process. In the context of working with refugees in the UK, Tribe [(1), p. 11] also endorses this view, suggesting that community-based mental health services “may prove more accessible, acceptable and relevant services which are more in line with other types of community care.” Besides these forms of services, there is also significant evidence to show that many people within culturally diverse communities are likely to utilize avenues other than professional therapists for dealing with mental distress, such elders in the community, religious leaders, priests, and traditional healers (19). These positive resources, including especially *traditional healing practices and systems* can be involved in the provision of mental health services through collaborations, partnerships, and community-based health systems. An example here is the Muthuswamy healing temple in India where research conducted by the National Institute of Mental Health and Neurological Sciences (NIMHANS), India concluded that people with mental health issues staying at the temple showed significant reduction in psychiatric rating scale scores. The researchers suggested that “[h]ealing temples may constitute a community resource for mentally ill people in cultures where they are recognized and valued… [and the] potential for effective alliances involving indigenous local resources needs to be considered” [(57), p. 40]. Similarly, Gone (33) points to the widespread use of talking circles, pipe ceremonies, sweat lodges and other culturally specific practices in the federal Indian Health Service in the United States to argue for a renewed focus on participation in traditional cultural practices, and attendant possibilities of spiritual transformations, shifts in collective identity and meaning making. Boksa et al. (37) also reiterate the centrality of local Indigenous knowledge as a guide to the development of relevant mental health systems. Mahony and Donnelly (44) also point out that spiritual and traditional healing practices can prove very useful in terms of promoting immigrant women's mental health.

Another way forward is to go beyond *cultural competence* frameworks and practice toward developing *cultural partnerships*. Cultural competence “refers to the awareness, knowledge, and skills and the processes needed by individuals, profession, organizations and systems to function effectively and appropriately in culturally diverse situations in general and in particular encounters from different cultures” [(3), p. 23]. Quite a few authors point to cultural competence as the most commonly used framework of practice in working with issues of mental health in culturally diverse settings (58–60). While the cultural competence framework has proved useful in terms of working across cultures, it suffers from a few significant flaws. Firstly, cultural competence frameworks approach culture from a purportedly value-neutral position, thereby ignoring the differences in power and the nature of historical and present-day oppression experienced by cultural groups (61). Secondly the “competence” approach focuses on the providers and their institutions and their capabilities to provide culturally appropriate services and disregards the participation of the clients and their communities (19). In circumstances where some cultural groups can be marginalized, as in the context of the issues of historical dispossession, racism, stereotyping, stigmatization, and power differentials, it becomes extremely important to work toward more equitable ways of engaging with communities (61–63). And finally, cultural competence draws on static notions of cultures that are not based on the reality of the constantly changing and transforming nature of cultures (61, 64).

These issues point toward the need for developing partnerships that are more equitable and that realign power relationships between service providers and individuals. The focus must be to move from traditional relationships built in power relationships to more interdependent and synergistic relationships (64, 65). A range of partnerships could be useful toward developing more effective mental health systems. They could include cultural partnerships between mental health providers and diverse cultural communities. It would certainly add to the nature of these partnerships if the providers also followed a deliberate policy of hiring workers of diverse backgrounds, and especially those from the communities that the service users come from. Murray and Skull (66) suggest that these forms of partnerships between refugee groups and health service providers have been shown to be more effective in terms of responding to health and other needs of the refugees than traditional top-down approaches. Partnerships could also be developed between mental health providers and traditional healers and/or community elders where synergies could be built on (54, 67). Finally, the relationship between the therapist and the client could be viewed as a cultural partnership, very much in line with the recovery approach, where the client would be an active participant in the process.

## Conclusion

In this article, some of the key considerations of working with diverse cultures in mental health have been explored and the point made that there can be severe repercussions on individuals and communities if systems and processes are not in place to enable mental health providers to work effectively across cultures. Each of these considerations in turn provides opportunities for new ways of engaging across cultures that can empower all parties involved rather than disempower and marginalize some groups while empowering others. Rather than approach the considerations from a deficit approach, where each of these is a problem, they can provide new avenues for developing integrated and holistic approaches toward working with mental health. A few of these avenues have been discussed in the paper, and some of these are already beginning to make inroads into mainstream mental health services, such as the emphasis on integrative services and the recovery approach. Others, which have been delineated in greater detail in this paper, such as working with positive resources in the community and cultural partnerships, are those where very small one-off projects have been embarked on and where arguably there is much more opportunity for broad based research and practice.

## Author contributions

The author confirms being the sole contributor of this work and approved it for publication.

### Conflict of interest statement

The author declares that the research was conducted in the absence of any commercial or financial relationships that could be construed as a potential conflict of interest. The reviewer BM and handling Editor declared their shared affiliation.
